# Evaluation of Pharmacological Rescue of Melanocortin-4 Receptor Nonsense Mutations by Aminoglycoside

**DOI:** 10.3390/life12111793

**Published:** 2022-11-05

**Authors:** Friederike Höpfner, Sarah Zschunke, Nanina Reininghaus, Iqra Sohail, Patrick Scheerer, Paolo Annibale, Heike Biebermann, Peter Kühnen

**Affiliations:** 1 Institute of Experimental Pediatric Endocrinology, Corporate Member of Freie Universität Berlin and Humboldt-Universität zu Berlin, Charité—Universitätsmedizin Berlin, Augustenburger Platz 1, 13353 Berlin, Germany; 2 Max Delbrück Center, Robert-Rössle-Straße 10, 13092 Berlin, Germany; 3 Group Protein X-ray Crystallography and Signal Transduction, Institute of Medical Physics and Biophysics, Charité—Universitätsmedizin Berlin, Corporate Member of Freie Universität Berlin and Humboldt-Universität zu Berlin, Virchowweg 6, 10117 Berlin, Germany; 4 DZHK (German Centre for Cardiovascular Research), Partner Site Berlin, Augustenburger Platz 1, 13353 Berlin, Germany; 5 School of Physics and Astronomy, University of St Andrews, North Haugh, St Andrews KY16 9SS, UK; 6 Institute of Pediatric Endocrinology, Charité—Universitätsmedizin Berlin, Corporate Member of Freie Universität Berlin and Humboldt-Universität zu Berlin, Augustenburger Platz 1, 13353 Berlin, Germany

**Keywords:** *melanocortin 4 receptor*, *MC4R*, stop mutation, PTC, translational readthrough, G418

## Abstract

The *melanocortin-4 receptor* (*MC4R*) is critical for central satiety regulation, therefore presenting a potent target for pharmacological obesity treatment. *Melanocortin-4 receptor* mutations prevalently cause monogenetic obesity. A possibility of overcoming stop mutations is aminoglycoside-mediated translational readthrough. Promising results were achieved in COS-7 cells, but data for human cell systems are still missing, so uncertainty surrounds this potential treatment. In transfected HEK-293 cells, we tested whether translational readthrough by aminoglycoside Geneticin combined with high-affinity ligand setmelanotide, which is effective in proopiomelanocortin or leptin receptor deficiency patients, is a treatment option for affected patients. Five *MC4R* nonsense mutants (W16X, Y35X_D37V, E61X, W258X, Q307X) were investigated. Confocal microscopy and cell surface expression assays revealed the importance of the mutations’ position within the *MC4R*. N-terminal mutants were marginally expressed independent of Geneticin treatment, whereas mutants with nonsense mutations in transmembrane helix 6 or helix 8 showed wild-type-like expression. For functional analysis, G_s_ and G_q/11_ signaling were measured. N-terminal mutants (W16X, Y35X_D37V) showed no cAMP formation after challenge with alpha-MSH or setmelanotide, irrespective of Geneticin treatment. Similarly, G_s_ activation was almost impossible in W258X and Q307X with wild-type-like cell surface expression. Results for G_q/11_ signaling were comparable. Based on our data, this approach improbably represents a therapeutic option.

## 1. Introduction

Obesity is an issue of global importance, affecting nearly 700 million people worldwide [1]. It is expected that in 2025, around 20% of the world’s population will suffer from obesity [2], which is associated with several comorbidities, such as diabetes, cardiovascular disease, infertility, and some cancer types, as well as increased mortality [3,4,5,6]. Often, overweight already develops in childhood, with over 340 million children aged 5–19 being overweight or obese in 2016, according to WHO [7]. Therefore, successful treating options for obesity are in high demand and urgently needed. However, obesity prevention and treatment have so far been unsuccessful in the long term, and especially several forms of obesity caused by monogenetic defects still lack treatment options [4,8,9]. The most common type of monogenetic obesity is linked to *melanocortin-4 receptor* (*MC4R*) deficiency, which was identified in 2–5% of obese patients [10].

*MC4R* is a G-protein-coupled receptor (GPCR) that physiologically plays a pivotal role in energy homeostasis, and its activation leads to decreased food intake [11]. Until now, the G_s_/adenylylcyclase pathway is seen as the major signaling pathway of *MC4R*. However, in recent years, it has become evident that *MC4R* can also signal via activation of a variety of pathways, such as G_q/11_ [12,13,14,15].

To date, over 300 *MC4R* mutations have been described [16,17]. Out of these, around 25% lead to a complete loss of function [18], causing hyperphagia with subsequent early-onset obesity [19,20,21]. Nonsense mutations, in which a premature termination codon (PTC) leads to early termination of translation and a nonfunctional protein, can occur in almost every part of the *MC4R*. Up until now, nonsense mutations have been identified at 22 different sites within the *MC4R* [17,18]. So far, no successful treatment option has been found for patients suffering from those mutations in *MC4R*.

A technique to overcome PTCs is translational readthrough, which was first mentioned in 1964 [22] and since then has been more broadly investigated. The goal of translational readthrough is to restore protein function, and it is a potential candidate for treating genetic diseases caused by PTCs [23]. It works by interfering with the process of translation. Readthrough agents increase the probability of continuation in translations by acting at the ribosome and purposefully inducing errors [24]. In this case, a deliberate error is wanted, as instead of the PTC, a random amino acid is inserted into the polypeptide, allowing for generating of a full-length protein.

Aminoglycoside antibiotics are very prominent readthrough agents, as discussed in an excellent review by Wang et al. [25]. Especially the aminoglycosides Geneticin (G418) and Gentamicin have been found to be good candidates for translational readthrough [26]. Cystic fibrosis was the first disease for which translational readthrough was considered as a new therapeutic approach, but several other genetic conditions have been investigated since, including Duchenne muscular dystrophy and nephrogenic diabetes insipidus [27,28,29].

For *MC4R* nonsense mutations, aminoglycoside-mediated translational readthrough may be an interesting therapeutic approach too. In this respect, some promising results have already been obtained in nonhuman CV-1 in Origin with SV40 gene (COS)-7 cells, transfected with various *MC4R* nonsense mutations (W16X, Y35X, E61X, Q307X). Treatment of COS-7 cells with aminoglycoside G418 led to an increase in full-length *MC4R* protein expression, and a partial regaining of functional properties of the investigated mutant, measured by intracellular cyclic adenosine monophosphate (cAMP) accumulation, could be restored to a certain extent [30]. To date, however, no data are available for a human cell model, so it is unclear whether translational readthrough is a potential treatment option for affected patients. Thus, in this study, we evaluated the hypothesis of a readthrough of stop mutations in combination with the synthetic *MC4R* ligand setmelanotide (SM) being a treatment option for obese patients carrying *MC4R* stop mutations and performed our investigation in a human cell model for the first time.

Therefore, we here used human embryonic kidney 293 (HEK-293) cells to come closer to the human physiological state than green monkey-derived COS-7 cells. HEK-293 cells are a standard cell system in GPCR research, which are readily available and allow a profound understanding about the function and regulation of GPCRs due to their overexpression of the latter [31]. It is noteworthy that HEK-293 cells have been found to hold some neuron-specific characteristics by expressing more than 60 neuronal genes [32,33]. This resemblance to neurons presents another advantage of using this specific cell model, as *MC4R* is mainly expressed in the paraventricular nucleus (PVN) of the hypothalamus [34]. Five different *MC4R* nonsense mutations were investigated (W16X, Y35X_D37V, E61X, W258X, Q307X), all of them positioned in different parts of *MC4R* (see Table 1), thus allowing for additionally inquiring the importance of the position of the respective mutation for its susceptibility to translational readthrough induced by aminoglycoside treatment.

Another important aspect of this study was the usage of a reliable assay to determine cell surface and total protein expression as well as the investigation of G_q/11_ signaling properties, which recently have been found to have a substantial effect on weight regulation [35], after G418 treatment. However, all previous research on the functional rescue of *MC4R* nonsense mutations has solely focused on G_s_ signaling, so this study may allow a new and more extensive perspective on this interesting issue.

## 2. Materials and Methods

### 2.1. Ligands and Plasmids

The ligand alpha melanocyte stimulating hormone (alpha-MSH) was purchased from Merck (Taufkirchen, Germany). MC4R-wild-type (WT) was amplified from genomic deoxyribonucleic acid (DNA) and cloned into eukaryotic expression vector pcDps (kindly provided by Torsten Schöneberg, University of Leipzig). Using site-directed mutagenesis, the *MC4R* mutations W16X, Y35X_D37V, E61X, W258X and Q307X were introduced into MC4R-WT. For confocal microscopy, the mutants were tagged with a yellow fluorescent protein (YFP) -tag at C-terminus. For protein expression measurements, MC4R-WT and mutations were cloned into pBiT3.1-N (Promega, Mannheim, Germany), yielding a N-terminally tagged receptor with the HiBiT protein tag.

### 2.2. Cell Culture

The HEK-293 cell line was purchased from ATCC. Cells were cultivated in minimal essential medium (MEM; Biochrom, Berlin, Germany) supplemented with 5% fetal bovine serum (FBS; Gibco, Carlsbad, CA, USA) and 1% nonessential amino acids (NEAA; Biochrom, Berlin, Germany) at 37 °C in humidified air containing 5% CO_2_. The HEK-293 cells were tested for mycoplasma contamination at regular intervals. For measurements of cAMP, cell viability, total, and cell surface expression as well as for reporter gene assays, 1.5 · 10^4^ cells per well were seeded in 96-well plates and incubated for 24 h. For viability and reporter gene assays, translucent 96-well plates (Falcon, Kaiserslautern, Germany) coated with poly-L-lysine (Gibco, Waltham, MA, USA) were used, and for cAMP and total and cell surface expression, white 96-well plates (Corning, Costar, NY, USA). For confocal microscopy, 1.5 · 10^5^ cells were seeded in translucent 6-well plates with one round 24 mm glass coverslip, #1.5, added to each well.

### 2.3. Transfection

Transient transfection of MC4R-WT and *MC4R* stop mutation plasmid DNA (0.45 ng/µL/well) was performed 24 h after the seeding of HEK-293 cells in supplement-free advanced MEM (Life technologies, Carlsbad, CA, USA). Metafectene (Biontex, Munich, Germany) was used as a transfection agent according to the manufacturer’s protocol. For confocal microscopy (FuGene HD, Promega, Mannheim, Germany) was used according to the manufacturer’s protocol. In case of antibiotic treatment, G418 (Roche Diagnostics GmbH, Mannheim, Germany) was added during transfection.

### 2.4. Antibiotic Kill Curve for Evaluation of Cell Viability

Colorimetric viability assay with CellTiter 96 AQ_ueous_ One Solution (Promega, Mannheim, Germany) was performed to determine the appropriate G418 concentration for the following assays [36]. Untransfected HEK-293 cells were treated with different concentrations of G418 (50–1000 µg/mL) and then incubated at 37 °C. After 48 h incubation, CellTiter 96^®^ AQ_ueous_ One Solution was added to each well following the manufacturer’s protocol, and cells were incubated for an additional 1.5 h. Measurements were performed with an Anthos microplate reader 2001 (Biochrom, Berlin, Germany). Controls with untreated cells as well as with 5 µg/mL puromycin were also conducted.

### 2.5. Confocal Microscopy

Cells were maintained in Opti-MEM (Gibco, Waltham, MA, USA), in the presence or absence of 125 µg/mL G418 and transfected with YFP-tagged *MC4R* stop mutations. As adapted from previous research [30], cells were incubated for 48 h at 37 °C. Then cells were washed three times with 1 mL phosphate-buffered saline (PBS) and, after incubation for 20 min at room temperature, fixated on a cover glass with 1 mL/well 4% paraformaldehyde (PFA). After 5 min incubation with 4′,6-diamidino-2-phenylindole (DAPI) at room temperature, cells were again washed three times with PBS before being embedded with a mounting medium (RotiMount FluorCare, Roth, Karlsruhe, Germany). Microscopic analysis of *MC4R* expression was performed with a confocal laser microscope (Leica DMi8 Leica Microsystems, Wetzlar, Germany), equipped with a White Light Laser and Hybrid Detectors. Excitation of YFP was performed using a 514 nm line. Images were edited and evaluated using the ImageJ software (v 1.53; National Institutes of Health, Bethesda, MD, USA).

### 2.6. Analysis of Cell Surface and Total Protein Expression

The Nano-Glo^®^ HiBiT detection system (Promega, Mannheim, Germany) was used to quantify the cell surface and the total expression of *MC4R* [12]. Measurements were performed according to the manufacturer’s protocol. Two days after transfection, the medium was changed to 50 µL/well Opti-MEM without phenol red, and 50 µL of either HiBiT extracellular substrate (Promega, Mannheim, Germany) or HiBiT lytic substrate (Promega, Mannheim, Germany) was added. After orbital shaking for 3 min at 300 cycles/min and incubation at room temperature for 10 min, luminescence was measured using a Berthold Microplate Reader (Mithras LB 940, Berthold Technologies GmbH & Co. KG, Bad Wildbad, Germany). HEK-293 cells transfected with the empty vector pcDNA3 served as background control.

### 2.7. Measurement of cAMP Increase via GloSensor^TM^

GloSensor™ assay enables measuring of G_s_ signaling via real-time measurement of cAMP formation [35]. HEK-293 cells were transfected with MC4R-WT or nonsense mutations. In case of antibiotic treatment, 125 µg/mL G418 was added to cells at the time of transfection. Cells without antibiotic treatment received MEM (Gibco, Waltham, MA, USA) only. Forty-eight hours after transfection, cells were equilibrated with a mixture of 88% CO_2_ dependent medium (Gibco, Waltham, MA, USA), 10% FBS, and 2% GloSensor™ cAMP reagent. Quantitative measurements of luminescence were performed using a plate reader (Mithras LB940, Berthold Technologies GmbH & Co., Bad Wildbad, Germany). Basal cAMP activity was measured for 10 min. Following stimulation of the cells with 1 µM alpha-MSH, or 1 µM SM, the cAMP accumulation was measured 21 times at 2 min intervals. GloSensor™ results were expressed as relative luminescence units (rlu). The total cAMP accumulation was assessed in a time-response curve. Quantification of the total cAMP accumulation was performed by calculating the area under the curve (AUC; Supplementary Information, Table S1).

### 2.8. Reporter Gene Assays for the Determination of PLC Activation

To draw conclusions about G_q/11_ signaling, phospholipase C (PLC) activation was assessed. Luciferase-based reporter gene assays were performed that use responsive elements in the promotor region of the gene encoding a firefly luciferase [37]. Equal amounts of MC4R-WT and *MC4R* stop mutations and nuclear factor of activated T-cells (NFAT) reporter plasmid were cotransfected. In the case of G418 treatment, 125 µg/mL G418 was added to cells at the time of transfection. After 48 h, cells were treated with alpha-MSH or SM, then incubated in supplement-free MEM at 37 °C with 5% CO_2_. After 6 h, the reaction was terminated by discarding the medium. Cells were lysed at room temperature using 50 µL passive lysis buffer (PLB; Promega, Fitchburg, WI, USA), then frozen at −80 °C for 10 min. Afterwards, 10 µL lysate was transferred onto a white opaque 96-well plate. Automatic injection of 40 µL firefly luciferase substrate (Promega, Mannheim, Germany) and determination of luminescence were performed with the plate reader Mithras LB940.

### 2.9. Statistical Analysis

Statistical testing and calculation of the AUC were performed using the GraphPad Prism 9.3.1 software (San Diego, CA, USA). The significance between parameters was calculated with one-way ANOVA (Dunnett’s test) and two-way ANOVA (Tukey’s test). *p* ≤ 0.05 was set as a significant outcome. All data represent means ± standard error of mean (SEM), if not indicated otherwise.

## 3. Results

### 3.1. Evaluation of G418 Cytotoxicity in HEK-293 Cells

To exclude significant cytotoxic side effects of aminoglycoside treatment, in a first step, the appropriate concentration of G418 was determined by cell viability assays in untransfected HEK-293 cells. In previous studies, the concentration of G418 used for readthrough experiments often ranges from 75 to 400 µg/mL [30,38,39,40]. As some of these studies have been performed in HEK-293 cells [39], but others in different cell lines [30,38,40], the appropriate concentration for the HEK-293 cells, which were used in our assays, had to be determined.

**Figure 1:**
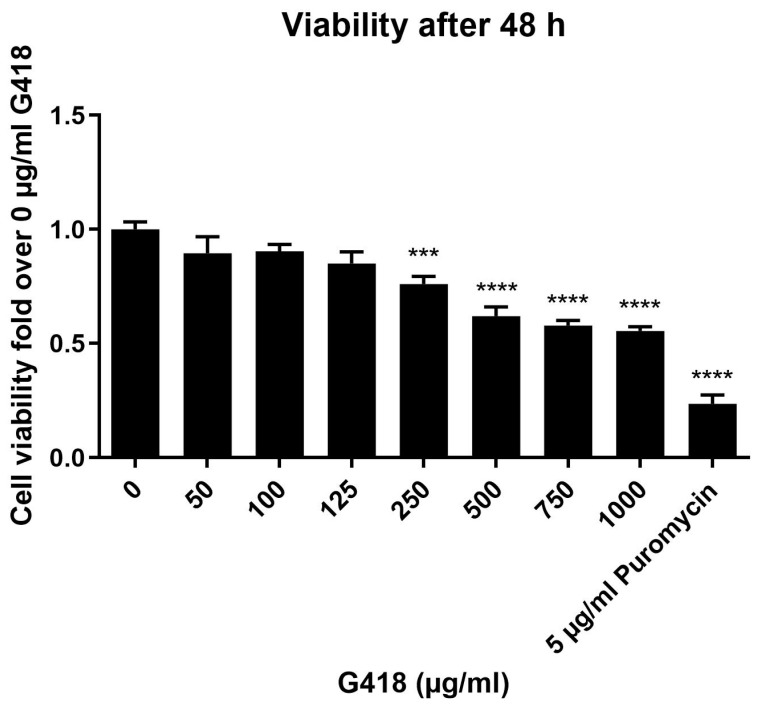
Concentration-dependent increase in the cytotoxicity of G418 in HEK-293 cells. Viability of cells was determined using untransfected HEK-293 cells and increasing concentrations of G418 (0–1000 µg/mL). Puromycin (5 µg/mL) served as a positive control. After incubation at 37 °C for 48 h, colorimetric measurements were performed (CellTiter A_Queous_ One Solution, Promega). The viability of cells with 0 µg/mL G418 was set at 1. With an increasing concentration of G418, a steady decrease in viability was seen. Data represent mean ± SEM from three independent experiments performed in triplicate. Significant differences were calculated using Dunnett’s test and are denoted as follows: *** *p*-value = 0.0001–0.001; **** *p*-value < 0.0001.

As shown in Figure 1, G418 (0–1000 µg/mL) concentration-dependently decreased the viability of HEK-293 cells by up to 45% after 48 h of treatment. However, significant cytotoxic effects became apparent only at concentrations of 250 µg/mL and higher. Up to 125 µg/mL, only a nonsignificant decrease of up to 15% occurred. The appropriate concentration for the functional assays was thus found to be 125 µg/mL, with two important factors playing into that decision: higher concentrations of G418 should yield more pronounced rescuing effects, whereas a good survival of the cells is vital for a safe treatment. This concentration is in accordance with previous studies [39].

Additionally, puromycin was used as a positive control for the cytotoxicity of aminoglycosides. Puromycin is known to be cytotoxic even at low concentrations [41,42], and accordingly, 5 µg/mL of puromycin already decreased the viability of HEK-293 cells by >75%.

### 3.2. Determination of *MC4R* Cell Surface and Total Expression

In a next step, the expression and localization of *MC4R* in transfected HEK-293 cells was determined using confocal microscopy and the HiBiT assay system.

#### 3.2.1. Confocal Microscopy Showed Successful Readthrough Activity

Confocal fluorescence microscopy was performed in the absence and presence of G418, where MC4R-WT and stop mutants were C-terminally tagged with YFP. W16X and Q307X, in which the mutations are positioned at different domains of the *MC4R*, are shown to be exemplary for the *MC4R* stop mutants.

**Figure 2:**
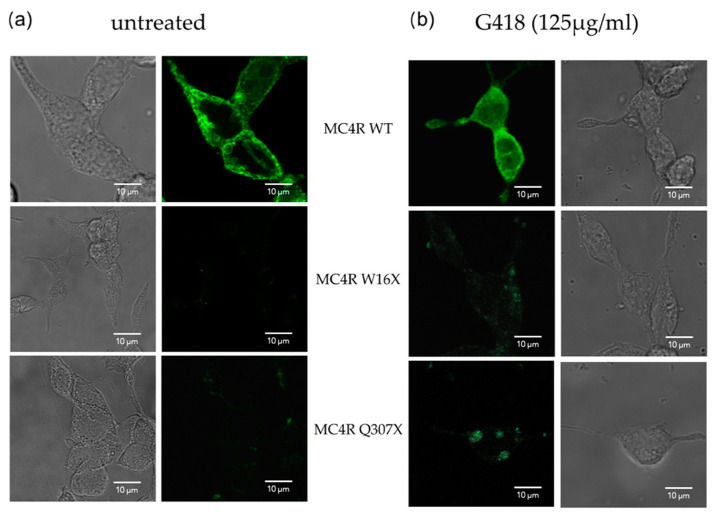
*MC4R* localization in the absence and presence of G418 is determined by confocal fluorescence microscopy. One column shows phase contrast microscopy, the other fluorescence microscopy with (**a**) depicting untreated and (**b**) G418 treated conditions. MC4R-WT and the stop mutants W16X and Q307X were tagged with YFP. The WT shows high expression on the cell surface and intracellularly with comparable results for the absence and presence of G418 (upper panel). *MC4R* stop mutants show very low signal in untreated conditions. After G418 treatment, a slight enhancement in signal is seen, but still to a lower degree than the WT. Contrast settings are the same in all fluorescence panels.

MC4R-WT showed a strong expression on the membrane and intracellularly (Figure 2, upper panel). *MC4R* expression is similar in both conditions, with or without G418. In contrast, for the *MC4R* nonsense mutants without G418, only a very faint signal could be detected (Figure 2, middle and lower panels). After G418 treatment, the signal was slightly enhanced, and distinct spots of *MC4R* could be detected, indicating that the readthrough is working. Compared with the WT, the fluorescence signal remained low for the *MC4R* stop mutants.

#### 3.2.2. HiBiT Assay for the Determination of Cell Surface and Total Receptor Expression

**Figure 3:**
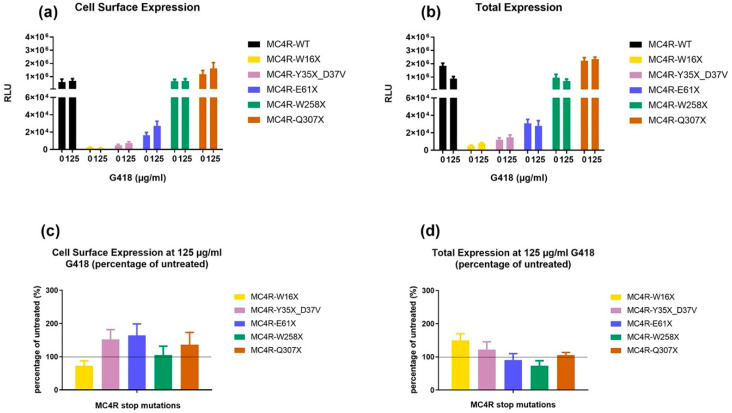
Expression of the *MC4R* variants in the absence or presence of G418. In the upper part, (**a**) shows cell surface expression, and (**b**) shows total expression of *MC4R*. In the lower segment, (**c**,**d**) show the percentage change of expression measured against their expression at untreated conditions. HEK-293 cells were transfected with N-terminally HiBiT-tagged MC4R-WT or mutation. This small protein tag is able to complement a split luciferase that cannot cross the plasma membrane. In case of antibiotic treatment, cells were incubated in medium containing 125 µg/mL G418. For cell surface expression (**a**), the cell membrane remained intact, and for the determination of total expression (**b**), the cells were lysed. The expression of the *MC4R* variant was quantified in rlu. Results are shown in pairs with the first column showing results of untreated cells and the second column showing the cells treated with G418. Calculation of the percentage change in the expression of *MC4R* stop mutants (**c**,**d**) showed that E61X had the highest increase regarding cell surface expression, whereas W16X had the highest increase in total protein expression. Values represent mean ± SEM from four independent experiments performed in triplicate.

Interestingly, the results of the HiBiT assay, which is dependent on the N-terminal HiBiT-tag, present differently compared with the microscopy data, which depend on a C-terminal fluorescent tag. The expression of MC4R-WT and mutant *MC4R* was detected on the cell surface (Figure 3a). Remarkably, cell surface expression strongly differed among the different *MC4R* mutants, and a certain pattern became apparent, indicating that cell surface expression was dependent on the position of the mutation within the GPCR: the earlier in the amino acid sequence the mutation occurs, the lower the expression on the cell surface is, indicating that an early stop codon abolishes receptor expression.

Accordingly, the W16X mutant, in which the PTC occurs in the N-terminus region of *MC4R*, showed a much lower cell surface expression than the WT. The Y35X_D37V mutant, which harbors mutations located only slightly further downstream the N-terminus, yielded very similar results. E61X with its PTC in the first transmembrane domain already showed a slightly higher cell surface expression, but nevertheless still to a much lower extent than the WT. On the other hand, the W258X mutant, in which the mutation is part of the highly conserved and important CWxP motif in *MC4R* and other GPCRs [37], in transmembrane helix (TMH) 6, and the Q307X mutant with a PTC in the beginning of helix 8 showed a cell surface expression that was similar to the WT. Comparable results were found when looking at the total expression of the receptor (Figure 3b).

To check for the putative effect of aminoglycoside treatment on the expression of mutated *MC4R*, G418 (125 µg/mL) was added during transfection. As shown in Figure 3c, the cell surface expression increased for all stop mutants except W16X. The effect was most pronounced in E61X. For total protein expression, Y35X_D37V showed the highest increase compared with untreated conditions. However, none of the results reached statistical significance.

### 3.3. G418 Did Not Restore Intracellular G_s_ Signaling of *MC4R* Stop Mutants

The functional properties of *MC4R* stop mutants challenged with the endogenous ligand alpha-MSH or the synthetic ligand SM and the modulation by G418 treatment were investigated by GloSensor^TM^ assays for the determination of G_s_ signaling. These allow the monitoring of intracellular cAMP accumulation in real time, thereby allowing the determination of G_s_ signaling properties of the *MC4R* variants.

**Figure 4:**
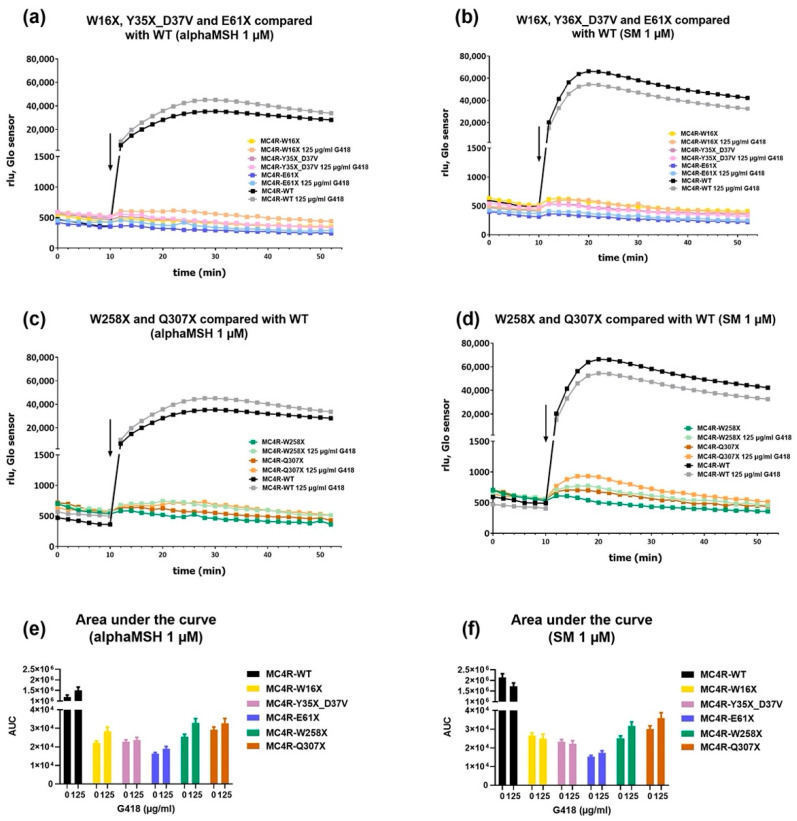
G_s_ signaling properties of *MC4R* variants after stimulation in the absence or presence of G418. HEK-293 cells were transfected with MC4R-WT or mutation and GloSensor™ reporter and incubated for 48 h, and in the case of antibiotic treatment, the medium contained 125 µg/mL G418. After a basal measurement, cells were stimulated with alpha-MSH (**a**,**c**) or SM (**b**,**d**), and cAMP accumulation was measured over time and quantified in rlu. The arrow indicates the start of stimulation. The WT showed a pronounced increase in cAMP accumulation after stimulation with both alpha-MSH and SM regardless of G418 treatment. While W16X, Y35X_D37V, and E61X showed very little to no increase in cAMP (**a**,**b**), stimulation of W258X and Q307X after G418 treatment led to a slightly more noticeable increase, especially when stimulated with SM (**c**,**d**). (**e**,**f**) show the increase in AUC after stimulation. Data represent three independent experiments, each performed in triplicate. Values represent mean (± SEM for (**e**,**f**)) from three independent experiments.

As expected, the stimulation of MC4R-WT with the endogenous ligand alpha-MSH (1 µM) led to an immediate and lasting increase in intracellular cAMP levels (Figure 4a). Stimulation with SM (1 µM) also increased the concentration of intracellular cAMP in a comparable manner, showing an even slightly higher induction than the activation with the endogenous ligand alpha-MSH (Figure 4b). In both cases, additional treatment with G418 (125 µg/mL) resulted in a similar cAMP induction, indicating that the cAMP signaling of fully functional MC4R-WT may not be affected by additional stimulation with aminoglycosides.

Almost no appreciable induction of cAMP was seen after stimulation of the mutants W16X, Y35X_D37V, and E61X with either the natural or the synthetic ligand regardless of G418 treatment (Figure 4c,d). Stimulation of the mutants W258X and Q307X with alpha-MSH (Figure 4e) or SM (Figure 4f) in the absence of G418 did not induce remarkable increases in intracellular cAMP levels. However, under G418 pretreated conditions, a more pronounced increase in cAMP was seen for both mutants, especially after stimulation with SM. Additional calculation of the AUC supported this notion, as the AUC of the mutants W258X and Q307X increased after G418 treatment (Figure 4g,h). Nevertheless, this increase by G418 treatment yielded no statistical significance, and in comparison with the AUC of the WT, both W258X and Q307X only were at ∼2% of the WT.

### 3.4. G418 Only Increased Basal G_q/11_ Signaling in *MC4R* Stop Mutants

To evaluate G_q/11_ signaling properties of *MC4R* nonsense mutants, reporter gene assays were performed, and PLC activity was measured through NFAT responsive element activity.

**Figure 5:**
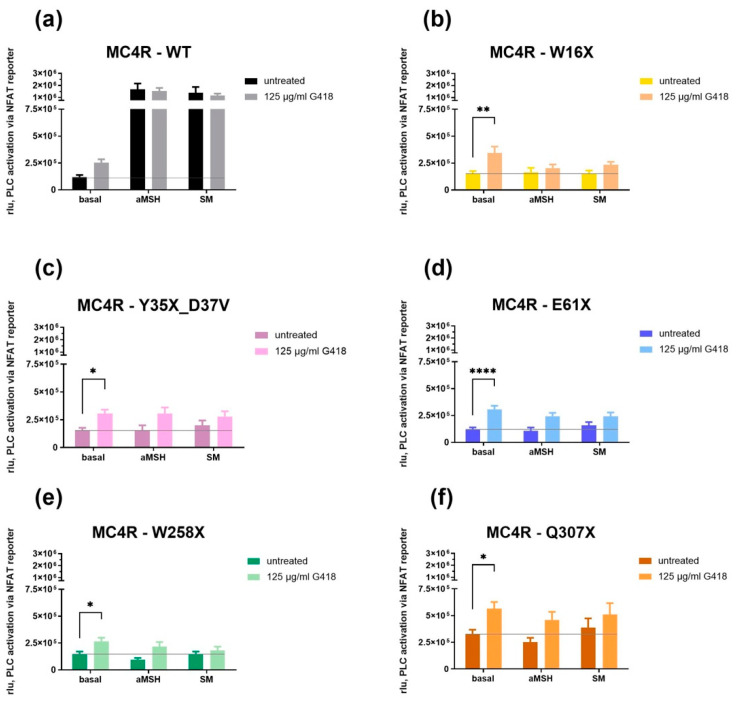
G_q/11_ signaling of MC4R-WT and mutants in the absence or presence of G418. HEK-293 cells were cotransfected with an *MC4R* variant and NFAT responsive element. In the case of antibiotic treatment, 125 µg/mL G418 was added to cells. Basal activity and activity after stimulation, with or without G418, was measured and quantified in rlu to determine G_q/11_ signaling capacities. Results of stimulation with alpha-MSH and SM are shown for MC4R-WT and each stop mutant separately (**a**–**f**). An increase in signaling after stimulation with both alpha-MSH and SM and both in the presence and absence of G418 was seen in the WT (**a**). In contrast, none of the mutants showed a significant increase in rlu after stimulation regardless of G418 treatment. However, a significantly higher basal activity after treatment with G418 could be measured in all *MC4R* mutants. Values represent mean ± SEM from four to eight independent experiments performed in triplicate. Significant differences (Tukey’s test) from the respective *MC4R* without aminoglycoside administration are denoted as follows: * *p*-value = 0.01–0.05; ** *p*-value = 0.001–0.01; **** *p*-value < 0.0001.

As anticipated, MC4R-WT showed a strong increase of signaling after stimulation with 1 µM alpha-MSH or SM (Figure 5a). A steady increase was measured both with and without G418 treatment, but the increase was slightly higher without G418 treatment. The basal activity of MC4R-WT was faintly increased after G418 treatment.

Interestingly, all *MC4R* stop mutants showed a significant increase in their basal activity after G418 treatment (Figure 5b–f). Nevertheless, stimulation with 1 µM alpha-MSH or SM did not result in G_q/11_ activation. Overall, PLC activation after stimulation was higher after G418 treatment for all *MC4R* stop mutants but not statistically significant.

In summary, functional data demonstrate that a readthrough of *MC4R* stop mutations in HEK-293 cells is generally possible; however, the efficacy in readthrough did not efficiently restore *MC4R* expression and function. So far, it is uncertain what the increase in basal activity in G_q/11_ signaling after G418 treatment means for *MC4R* function in vivo. Therefore, at this point, we have to conclude that readthrough of *MC4R* stop mutations does not represent a promising treatment strategy.

## 4. Discussion

The *MC4R* is a key player in energy homeostasis and satiety regulation [12], and *MC4R* deficiency is the most common cause of monogenetic obesity [35]. It therefore is an interesting target for antiobesity treatment approaches. SM is a high-affinity ligand for *MC4R*, described in 2021 as an MC4R-SM structure complex [37], and has been approved in 2020 by the U.S. Food and Drug Administration (FDA) for the treatment of some forms of rare monogenetic obesity due to mutations in the melanocortin–leptin pathway, namely, pro-opiomelanocortin (*POMC*) deficiency, proprotein subtilisin/kexin type 1 (*PCSK1*) deficiency, and leptin receptor (*LEPR*) deficiency [43]. However, up until today, no treatment options exist for patients suffering from *MC4R* deficiency due to *MC4R* nonsense mutations. Recently, the efficacy of glucagon-like peptide (GLP)-1 receptor agonist treatment has been evaluated in short-term clinical studies [44]. Whereas incretin treatment targets the GLP-1 receptor, for stop mutations directly affecting the *MC4R*, aminoglycosides have been shown to effectively induce the readthrough of PTCs. As PTCs usually lead to truncated and functionless proteins, aminoglycoside-induced readthrough restores the expression and functional properties of proteins [38] and may thus also work in the restoration of the expression and function of nonsense mutated *MC4R*.

The use of translational readthrough agents has been investigated in several clinical studies, including a trial on the use of Gentamicin in recessive dystrophic epidermolysis bullosa (RDEB). The conclusion of their study was that Gentamicin therapy might be a readily available treatment option for patients suffering from RDEB due to nonsense mutations [45]. Additionally, since 2014, the drug Translarna, a nonaminoglycoside drug that induces translational readthrough, has been approved by the European Medicines Agency (EMA) as an orphan drug for the treatment of Duchenne muscular dystrophy resulting from a nonsense mutation in the dystrophin gene in ambulatory patients aged 2 years and older [46]. More closely related to our research, the rescue of nonsense mutated leptin receptors causing monogenetic obesity was attempted in an in vitro setting using HEK-293 cells and the readthrough agents G418, Gentamicin, and ataluren. Surprisingly, the investigated human nonsense mutation could be suppressed when characterized within nonhuman receptors, but not after insertion into the human receptor [47].

Thus, we here evaluated whether treatment with the aminoglycoside G418 can rescue *MC4R* stop mutations and increase receptor expression as well as signaling in human MC4R-transfected HEK-293 cells. Functional testing of G_s_ and G_q/11_ signaling of the receptors was performed after challenging them with the endogenous *MC4R* ligand alpha-MSH as well as with the synthetic cyclic ligand SM, which has also been shown to be an interesting candidate for the therapeutic activation of *MC4R* mutations [8].

We conducted research on human HEK-293 cells transiently transfected with different *MC4R* stop mutations, as so far, a rescue of *MC4R* stop mutations has only been investigated in nonhuman COS-7 cells, focusing on G_s_ signaling only, and the results were auspicious [30]. In our study, we first wanted to use a human cell system and, second, also get a broader overview by investigating not only Gs signaling but also G_q/11_ signaling, which since then has been found out to be of importance.

After determining a nontoxic and appropriate concentration of G418 in viability assays, MC4R-WT and five selected *MC4R* stop mutants (W16X, Y35X_D37V, E61X, W258X, Q307X) were investigated for their expression and signaling properties after treatment with 125 µg/mL G418, and the findings were compared with untreated control conditions. Surprisingly, HiBiT assays and confocal microscopy, both focusing on the detection of *MC4R* expression and localization patterns, revealed results that seemed to contradict each other at first: Using confocal fluorescence microscopy, hardly any expression of *MC4R* stop mutants could be detected in untreated conditions, which is expected. After G418 treatment, the expression appeared higher, but still low compared with the WT. The reason for this might be that by readthrough, a random amino acid is incorporated, probably resulting in misfolding of the receptor in case the position of the *MC4R* stop mutation is of importance for receptor conformation. In the HiBiT cell surface assay, however, the expression of *MC4R* stop mutants varied to a great extent, and the *MC4R* stop mutants with PTCs further downstream (W258X, Q307X) showed a WT-like expression, because here, correct folding and membrane expression might occur, while the early N-terminal mutant (W16X) had by far the lowest expression, with or without G418.

We can therefore assume that *MC4R* is expressed on the cell surface despite the stop mutation, especially if the mutation occurs further downstream of the receptor. These results contradict the previous finding of Moore et al. from 2018, which state that truncation early in the *MC4R* C-terminus (before position C318) leads to improper localization and no signaling [48]. We detected *MC4R* stop mutants on the cell surface, with especially W258X and Q307X showing WT-like expression. However, in the case of the Q307X mutant, parts of helix 8 and the remaining C-terminus are missing due to the early termination.

This interesting finding might be explained by the fact that *MC4R* does not induce nonsense-mediated decay (NMD). NMD describes a process in which ribonucleic acid (RNA) selectively gets degraded after a PTC is detected in its sequence [49]. Thus, this surveillance mechanism is supposed to remove RNA, which would produce a protein with a harmful effect for the organism [50]. In mammals, NMD is dependent on exon–exon junctions after post-translational splicing. However, as the *MC4R* is a single exon gene, no exon–exon junction exists, and therefore, *MC4R* mutants harboring a PTC are insensitive to NMD [51].

As after G418 treatment some improvement was seen in *MC4R* expression, we wanted to determine whether a rescue of functional properties was possible. In COS-7 cells, it has been shown that a readthrough of nonsense mutations is generally possible, and even some functional rescue was possible [30]. At first, we performed GloSensor^TM^ assays to enable live measurement of cAMP accumulation and, therefore, deduce G_s_ signaling capacities. In the presence of G418, cAMP accumulation of stimulated *MC4R* stop mutants was only slightly and not significantly increased. This is concordant with the findings from our HiBiT assays, in which protein expression is slightly but not significantly increased for most *MC4R* stop mutants after G418 treatment.

As in recent years, the role of G_q/11_ has been found to be of importance for *MC4R* signaling [12,13,14,15], an investigation about a possible rescue of this pathway was compelling. Interestingly, our results showed a significant increase in basal G_q/11_ signaling after G418 treatment for all stop mutants, but no G_q/11_ activation after stimulation was observed. This is a new and intriguing finding, but so far, the effect this might have in vivo remains unclear.

Overall, G418 was not able to restore substantial improvement of G_s_ and G_q/11_ signaling in HEK-293 cells. Additionally, results obtained in nonhuman COS-7 cells concerning the rescue of *MC4R* stop mutations could not be replicated in the human cell model. This might be due to certain differences between the cell lines, such as the difference in the expression of regulatory proteins. Melanocortin 2 receptor accessory protein 2 (MRAP2) is a regulatory protein that is expressed in the HEK-293 cells we used [52], but not in COS-7 cells [53]. Several studies have shown that *MC4R* signaling is highly affected by MRAP2 [54,55,56,57,58]; therefore, the presence or rather, in the case of COS-7 cells, the absence of MRAP2 can affect results. Furthermore, the SV40 promoter used for our *MC4R* expression plasmids is ideal for COS-7 cells [59] but results in reduced expression in HEK-293 cells, which is wanted in our study to better resemble the physiological state. These findings could also explain the observed differences. It is noteworthy that for the leptin receptor, similar results were obtained in the aspect that aminoglycoside-mediated rescue only worked in a nonhuman setting [47].

Some distinctions can be found between the five investigated *MC4R* nonsense mutations: The surrounding nucleotides vary as well as the respective stop codon sequence, as shown in Table 1. In previous studies, these differences have been found to be of importance in terms of the success of aminoglycoside-mediated readthrough [30].

G418 has been shown to have a comparable readthrough activity to all three PTCs, but slightly higher for TAG and TGA than for TAA [60]. In our HiBiT assay, W16X, which carries the TGA codon, showed the highest increase in total protein expression after G418 treatment. Additionally, W16X and E61X, which carry the TAG codon, had the most significant increase in basal activity after G418 treatment in the NFAT assay. However, a stimulation of neither G_s_ nor G_q/11_ signaling was remarkably different from the *MC4R* stop mutants Y35X_D37V and Q307X, which harbor the TAA codon.

Besides the sequence of the stop codon, the nucleotide directly following the stop codon is known as another factor to play a role in the success of readthrough. Apparently, translational readthrough is most successful if cytosine or adenine is incorporated as the next nucleotide [30]. Again, this is the case for W16X, with adenine in the following position. E61X also has adenine as the nucleotide following the stop codon. In HiBiT assays, E61X showed the highest increase in the cell surface expression of *MC4R* and the most significant increase in basal activity in the NFAT assays.

Certainly, the amino acid that is inserted into the sequence during translational readthrough can also affect the formation of the peptide. As this is a random process, it cannot be interfered with. However, some positions within the *MC4R* are less conserved than others, which indicates that these positions are less important for receptor activity than others. For example, the N-terminus is not conserved throughout different species [61]; therefore, it is likely that the insertion of a random amino acid within the N-terminus is affecting the receptor less than in other parts. However, in our functional assays, we could find no such difference between the two N-terminal mutants W16X and Y35X_D37V compared with the other mutants with PTCs occurring further downstream, as significant induction of signaling was not possible for any of the mutants. Any effect was probably more related to reduced expression than to the position of the mutation.

In conclusion, based on our findings and the currently lacking knowledge on the importance of basal G_q/11_ signaling, we have to state that G418 treatment in combination with SM is very unlikely to be useful as a new therapeutic approach. However, our cell model, albeit already much closer to the physiological state than kidney cells of green monkeys like COS-7 cells, is not ideal, as HEK-293 are not neuronal cells, and it is an in vitro study. It would be intriguing to investigate translational readthrough of the *MC4R* functions in a human neuronal cell line or even in an in vivo setting. The in vivo setting might help clarify the increased basal activity in our NFAT assays and its consequences for the organism. It could also be interesting to focus on the mutations, which have a high potential of functional rescue due to their position within the receptor or their surrounding nucleotides, such as W16X. In COS-7 cells, G418 showed the best readthrough activity compared with other aminoglycosides [30]. As we now found different results in HEK-293 cells compared with COS-7 cells, it might also be useful to test the efficacy of other readthrough agents on HEK-293 cells. These could be different aminoglycosides or also other nonaminoglycoside readthrough-inducing agents, of which many have been developed or investigated in recent years [60,62,63].

## Figures and Tables

**Table 1:** Analyzed *MC4R* stop mutations and their stop codon sequences.

*MC4R* Mutation	WT Sequence	Stop Mutation Sequence	Stop Codon Name	Localization of Mutation
W16X	CAC-CTC-**TGG**-AAC-CGC	CAC-CTC-**TGA**-AAC-CGC	opal	N-terminus
Y35X_D37V	AAA-GGC-**TAC**-TCT-**GAT**	AAA-GGC-**TAA**-TCT-**GTT**	ochre	N-terminus
E61X	TTG-TTG-**GAG**-AAT-ATC	TTG-TTG-**TAG**-AAT-ATC	amber	TMH 1
W258X	GTC-TGC-**TGG**-GCC-CCA	GTC-TGC-**TGA**-GCC-CCA	opal	TMH 6
Q307X	CGG-AGT-**CAA**-CAA-CTG	CGG-AGT-**TAA**-GAA-CTG	ochre	helix 8

## Data Availability

Data are contained within the article or supplementary material. The data presented in this study are available in Table S1, “Source data file”.

## Supplementary Materials

The following supporting information can be downloaded at: https://www.mdpi.com/article/10.3390/life12111793/s1, Table S1: Source data.
